# Epileptiform activity in the acute phase of stroke predicts the outcomes in patients without seizures

**DOI:** 10.3389/fneur.2023.1096876

**Published:** 2023-03-13

**Authors:** Anetta Lasek-Bal, Milena Dewerenda-Sikora, Łukasz Binek, Sebastian Student, Beata Łabuz-Roszak, Ewa Krzystanek, Aleksandra Kaczmarczyk, Aleksandra Krzan, Amadeusz Żak, Aleksandra Cieślik, Magdalena Bosak

**Affiliations:** ^1^Department of Neurology, School of Health Sciences, Medical University of Silesia, Katowice, Poland; ^2^Department of Neurology, Upper-Silesian Medical Centre of the Silesian Medical University, Katowice, Poland; ^3^Faculty of Automatic Control Electronics and Computer Science, Silesian University of Technology, Gliwice, Poland; ^4^Biotechnology Center, Silesian University of Technology, Gliwice, Poland; ^5^Department of Neurology, Institute of Medical Sciences University of Opole, Opole, Poland; ^6^Department of Neurology, Faculty of Medical Sciences in Katowice, Medical University of Silesia, Katowice, Poland; ^7^Department of Neurology, Jagiellonian University Medical College, Krakow, Poland

**Keywords:** EEG, brain activity, stroke, Rankin, epileptiform abnormalities

## Abstract

**Background and purpose:**

The abnormalities in EEG of stroke-patients increase the risk of epilepsy but their significancy for poststroke outcome is unclear. This presented study was aimed at determining the prevalence and nature of changes in EEG recordings from the stroke hemisphere and from the contralateral hemisphere. Another objective was to determine the significance of abnormalities in EEG in the first days of stroke for the post-stroke functional status on the acute and chronic phase of disease.

**Methods:**

In all qualified stroke-patients, EEG was performed during the first 3 days of hospitalization and at discharge. The correlation between EEG abnormalities both in the stroke hemisphere and in the collateral hemisphere with the neurological and functional state in various time points was performed.

**Results:**

One hundred thirty-one patients were enrolled to this study. Fifty-eight patients (44.27%) had abnormal EEG. The sporadic discharges and generalized rhythmic delta activity were the most common abnormalities in the EEG. The neurological status on the first day and the absence of changes in the EEG in the hemisphere without stroke were the independent factors for good neurological state (0–2 mRS) at discharge. The age-based analysis model (OR 0.981 CI 95% 0.959–1.001, *p* = 0.047), neurological status on day 1 (OR 0.884 CI 95% 0.82–0.942, *p* < 0.0001) and EEG recording above the healthy hemisphere (OR 0.607 CI 95% 0.37–0.917, *p* = 0.028) had the highest prognostic value in terms of achieving good status 90 days after stroke.

**Conclusions:**

Abnormalities in EEG without clinical manifestation are present in 40% of patients with acute stroke. Changes in EEG in acute stroke are associated with a poor neurological status in the first days and poor functional status in the chronic period of stroke.

## Introduction

The prevalence of epileptic seizures among acute stroke patients is reported to be in the range of 4–10% and is considered an unfavorable prognostic factor regarding the post-stroke functional status (1–4). The severity of a patient's neurological status during acute stroke period, as well as the size of infarct focus, large-artery atherosclerotic etiology, early seizures, cortical involvement, and middle cerebral artery involvement are known risk factors for epileptic seizures and vascular epilepsy (4–6). Early changes in the electroencephalogram (EEG) of stroke patients have been shown to increase the risk of epilepsy within 1 year from stroke onset; the prognostic value of EEG in this respect exceeds the value of neuroimaging (7). There are also the contrary observations (3).

According to several studies, epileptiform abnormalities found in the EEG of patients with intracranial hemorrhage negatively impacts their functional status (8, 9).

EEG is a common, mostly non-invasive diagnostic method used to assess various functional changes in the brain. Despite the rapid development of advanced neuroimaging techniques, EEG remains an important supplementary examination for the investigation of neurological and non-neurological disorders and provides valuable and accurate information concerning brain function. EEG is particularly useful in the diagnosis and monitoring of patients with seizure. It is of great use in regards to the diagnosis of epilepsy as well as the classification of seizures/syndromes and can even provide some information in terms of prognosis in some cases.

An EEG reading is also incredibly useful for investigating other neurological disorders such as dementia, cerebrovascular diseases, toxic or metabolic encephalopathy, viral and autoimmune encephalitis, coma and brainstem death, among others. An EEG may also be helpful in a differential diagnosis of psychiatric and metabolic disorders, intoxications, alterations of consciousness, and many others.

Ischemic stroke patients have also shown a correlation between abnormal EEG results (especially electrographic epileptiform abnormalities ≥1.5 Hz) and poorer functional status (10, 11). However, the patients studied in the presented undertaking mainly experienced epileptic seizures during the acute stroke period and/or were taking anti-epileptic medication. Changes in the EEG associated with infarct focus may also turn out to be important for post-stroke prognosis in patients without epileptic seizures.

The majority of acute stroke foci, even those located in the cerebral cortex, do not cause clinical epileptic seizures during the acute stroke period. The significance of abnormal EEG recordings in patients with acute ischemic stroke and no epileptic seizures has only been studied occasionally (12, 13).

Also of interest is the potential relevance of thrombolytic therapy to the risk of seizure activity in the EEG and the risk of epileptic seizures. Some authors have found a higher risk of epileptic seizures in stroke patients treated with thrombolytic therapy (14, 15). The significance of thrombectomy in this regard is not clear (16).

This presented study was aimed at determining the prevalence and nature of changes in EEG recordings from the stroke hemisphere and from the contralateral hemisphere. Another objective was to determine the significance of abnormalities in EEG in the first days of stroke for the post-stroke functional status on the acute and chronic phase of disease.

## Methods

A prospective study between 2019 and 2021 looked at patients hospitalized in two Departments of Neurology in Katowice due to their first-ever symptomatic ischemic stroke (complete or reversible ischemic neurological deficit) diagnosed according to WHO criteria. The study excluded patients with hemorrhagic transformation of acute ischemic lesions, transient ischemic attack, acute multifocal stroke and patients with previous symptomatic stroke. Additional exclusion criteria were: dementia, brain tumor, epilepsy, seizures during hospitalization, discharge after 14 days and treatment with anti-epileptic drugs due to epilepsy or other indications.

The following parameters were analyzed for the participants enrolled in the study:

- Age at which the first ischemic stroke occurred.
- Type of ischemic stroke according to OCST.
- Comorbidities such as arterial hypertension, atrial fibrillation (AF), diabetes mellitus (DM), lipid disorders (LD), heart failure (HF).
- Neurological state during the first 24 and 48 h of stroke according to NIHSS (National Institutes of Health Stroke Scale).
- Functional status according to the modified Rankin Scale (mRS) at discharge and on day 90.
- EEG performed two times during the first 3 days and 1 day before discharge (the first EEG was performed on the 2^th^ or the 3^th^ day of hospitalization and the second one on the 12^th^ or 13^th^ day of hospitalization).

We defined epileptiform abnormalities (EAs) as sporadic epileptiform discharges, electrographic and electroclinical seizures, electrographic and electroclinical status epilepticus according to ACNS Critical Care EEG Terminology: 2021 Version (17).

### Description of the EEG procedure

A Galileo EEG-EP device from EB Neuro, equipped with 21 electrodes, was used for examinations. Scalp EEGs were recorded according to a standard 20-min clinical protocol including hyperventilation (Hv) and photostimulation (Fs). The examination was conducted at rest, in a supine position. EEG electrodes were placed on the patient's scalp in the conventional 10–20 system. A longitudinal bipolar montage channel configuration was used to collect the signals. The EEG was qualitatively analyzed by a single neurologist EEG-certificated by Polish Association of Neurology, blinded on the patient's neurological state and the neurological data during the EEG-analysis.

### Description of statistical methods

Parametric variables were characterized using the mean, standard deviation, median, minimum, and maximum values. Non-parametric variables were described by numbers and percentages represented in the study group. Comparisons between groups used the following methods for parametric variables: a Student's *T*-test was implemented for separable variables in the case of any variables with normal distribution; a Mann–Whitney U test for any variables which failed to meet the criteria for normal distribution. The normality of the data was checked with the Shapiro-Wilk test and visual observation of the Q-Q plot to check whether a data transformation was needed. Normality testing was done before the selection of the appropriate inferential statistical method. The *p* < 0.05 was considered significant. Any non-parametric variables were compared using the Chi-square test. Multiple significance testing corrections were used based on the Benjamini-Hochberg procedure (18). The Benjamini-Hochberg method was implemented in the R stats package using the FDR-adjusted *p*-value. The FDR adjusted *p*-value was calculated using the formula (m/i)^*^raw_p_value, where “m” was the total number of tests and “i” was the rank of the covariate by *p*-value. The FDR adjusted *p* < 0.05 was considered significant (19).

Multivariate models were built using ordinal logistic regression for ordinal outcomes. The model variable selection procedures included automatic parameter selection (stepwise, forward, and backward) based on the Akaike Information Criteria (AIC). The reduced model included only the best parameters and the highest AIC scores. To evaluate the accuracy of model predictions, the “leave one out” procedure was used to avoid data leakage so as not to cause over-fitting (Tasman 2000); multiclass AUC (mcAUC) estimators were also used (Hand 2001). All statistical analyzes were performed with R version 4.0.5 (18).

The study was accepted by the Ethics Committee of the Silesian Medical University of Silesia in Katowice.

## Results

We enrolled 131 patients treated for stroke between 1 Jan 2019 and 31 Dec 2021 at two academic Neurology Centers in Katowice, Poland, including 69 women (52.65%). The mean age of the patients was 69.35 ± 3.21, med. 70. The median admission NIHSS score was 6. Most of the patients had PACI (partial anterior cerebral ischemia) (85; 81.73%).

Fifty-eight patients (44.27%) showed changes in EEG at least in one examination.

Patients with abnormal EEG results were significantly older than those without changes in EEG. In addition to that, there were no significant differences in the burden of comorbidities, stroke types (PACI was predominant in both groups) or therapy types. Table 1 presents the clinical characteristics of the patients and a comparison of the study groups divided by EEG recordings.

**Table 1:** The characteristic of the stroke patients with and without changes in the EEG.

**Parameters**	**All patients *n* = 131**	**Patients with abnormal EEG readings*n* = 58**	**Patients without abnormalities in the EEG *n* = 73**	**FDR-adjusted *p*-value(EEG+ patients vs. EEG- patients)**
Age, mean, med. [range] [IQR]	69.35; 70; [73] [17.5]	72.55; 74.5; [59] [16.5]	66.81; 67; [68] [17]	0.02
F/M	69/62 52.65%/47.33%	37/2163.79%/36.21,	32/41 43.84%/56.16%	0.07
OCSP, *n* (%)PACIPOCILACITACI	85 (81.73%) 16 (15.38%) 0% 3 (2.88%)	84.85%9.09%0%6.06%	80.28% 18.31% 0% 1.41%	0.36
Admission NIHSS, med. [range] [IQR]	6; [25] [7]	8; [24] [9]	4; [20] [5]	<0.001
DM	37	16 (27.58%)	21 (28.76%)	0.44
AF	24	11 (18.96%)	13 (17.80%)	0.56
LD	30	13 (22.41%)	17 (23.28%)	0.32
HF	22	10 (17.24%)	12 (16.43%)	0.51
CHA_2_DSVA_2_SC, mean, med. [range] [IQR]	5.5; [8] [2]	5.140; 5; [6] [2]	4.890; 5; [8] [2]	0.29
Therapy, *n* (%)rtPAMTrtPA+ MTOther	26.92% 12.5% 9.62% 50.96%	21.21%15.15%9.09%54.55%	29.58% 11.27% 9.86% 49.30%	0.85

AF, atrial fibrillation; DM, diabetes mellitus; EEG, electroencephalogram; F, female; HF, heart failure; LACI, lacunar circulation infarcts; LD, lipid disorders; M, male; MT, mechanical thrombectomy; OCSP, Oxfordshire Community Stroke Project; PACI, breaktial anterior circulation infarcts; POCI, posterior circulation infarcts; rtPA, recombinant tissue, plasminogen activator; TACI, total anterior circulation infarcts.

Among patients with left hemisphere stroke 56.49% had the EAs as well as 33.59% patients with right hemisphere stroke and 8.4% with non-hemispheric stroke. In the group of patients with EAs 63.79% had the left hemispheric stroke, 32.76% right hemispheric stroke and the rest suffered with non-hemispheric stroke.

Fifty-two patients (39.69 %) scored >2 on the mRS on the day of discharge, including one patient who died during hospitalization. On day 90 following onset, 30 patients (22.9%) scored a maximum of 2 points on the mRS.

Sporadic discharges and generalized rhythmic delta activity (GRDA) were the most common abnormalities observed in the EEG results.

GRDA was located primarily in the stroke hemisphere. The predominance of such abnormalities coming from an area above the stroke hemisphere were observed in examinations repeated after some days. GRDA was the most common abnormality in EEG results recorded in the contralateral hemisphere.

Table 2 presents the types of changes found during EEG examinations with a percentage breakdown.

**Table 2:** Changes in the EEG in the stroke hemisphere and in the contralateral hemisphere.

	**Stroke hemisphere, *n* (%) patients**,	**Contralateral hemisphere, *n* (%) patients**
**EEG patterns in the 1st EEG reading** ***n*** **(%)**		
Lateralized periodic discharges	14 (10.68)	2 (1.52)
Generalized periodic discharges	10 (7.63)	10 (7.63)
Lateralized rhythmic delta activity	15 (11.45)	4 (3.05)
Focal irregular delta activity	8 (6.10)	3 (2.29)
Generalized rhythmic delta activity	19 (14.50)	19 (14.50)
Generalized irregular delta activity	3 (2.21)	0 (0)
Sporadic discharges	20 (15.26)	9 (6.80)
Electrographic seizures	18 (13.70)	1 (0.76)
**EEG patterns in the 2nd EEG reading**		
Lateralized periodic discharges	0 (0)	6 (4.58)
Generalized periodic discharges	8 (6.10)	8 (6.10)
Lateralized rhythmic delta activity	16 (12.21)	11 (8.39)
Focal irregular delta activity	6 (4.58)	2 (1.52)
Generalized rhythmic delta activity	19 (14.50)	19 (14.50)
Generalized irregular delta activity	1 (3.22)	0 (0)
Sporadic discharges	22 (16.79)	8 (6.90)
Electrographic seizures	14 (10.68)	0

EEG, electroencephalogram.

Multiple patterns were observed in 21 patients (36.20%).

The neurological status of patients with abnormal EEG recordings was significantly worse (according to NIHSS) on days 1 and 2 of stroke than with patients with normal EEG recordings; the functional status of patients with abnormal EEG recordings was significantly worse on the day of discharge and 90 days after the onset than that of patients with normal EEG recordings (Table 3).

**Table 3:** The neurological and functional status of stroke patients.

**Parameters**	**All patients *n* = 131**	**Patients with abnormal EEG recordings *n* = 58**	**Patients without abnormalities in the EEG *n* = 73**	**FDR-adjusted *p*-value (EEG+ patients vs. EEG- patients)**
NIHSS 1, med. [range] [IQR]	6; [25] [7]	8; [24] [9]	4; [20] [5]	<0.01
NIHSS 2, med. [range] [IQR]	2; [0.21] [5]	4; [21] [6.75]	1; [10] [2]	<0.01
ΔNIHSS (at least 4 points less), n (%)	45 (34.62)	33 (57.58)	50 (69.01)	0.48
mRS, (discharge), med. [range] [IQR]	2; [0.60] [3]	3; [6] [2]	1; [4] [3]	<0.01
mRS (after 3 months), med. [range] [IQR]	1; [0.60] [3]	3; [6] [2.75]	1; [5] [2]	<0.01

EEG, electroencephalography; EEG+, patients with abnormalities in EEG; EEG-, patients without EEG- abnormalities; NIHSS 1, National Institutes of Health Stroke Scale in the days 1-3 of stroke; mRS, modified Rankin Scale; NIHSS 2, National Institutes of Health Stroke Scale in at discharge; ΔNIHSS, the difference between NIHSS_1 and NIHSS_2.

**Figure 1:**
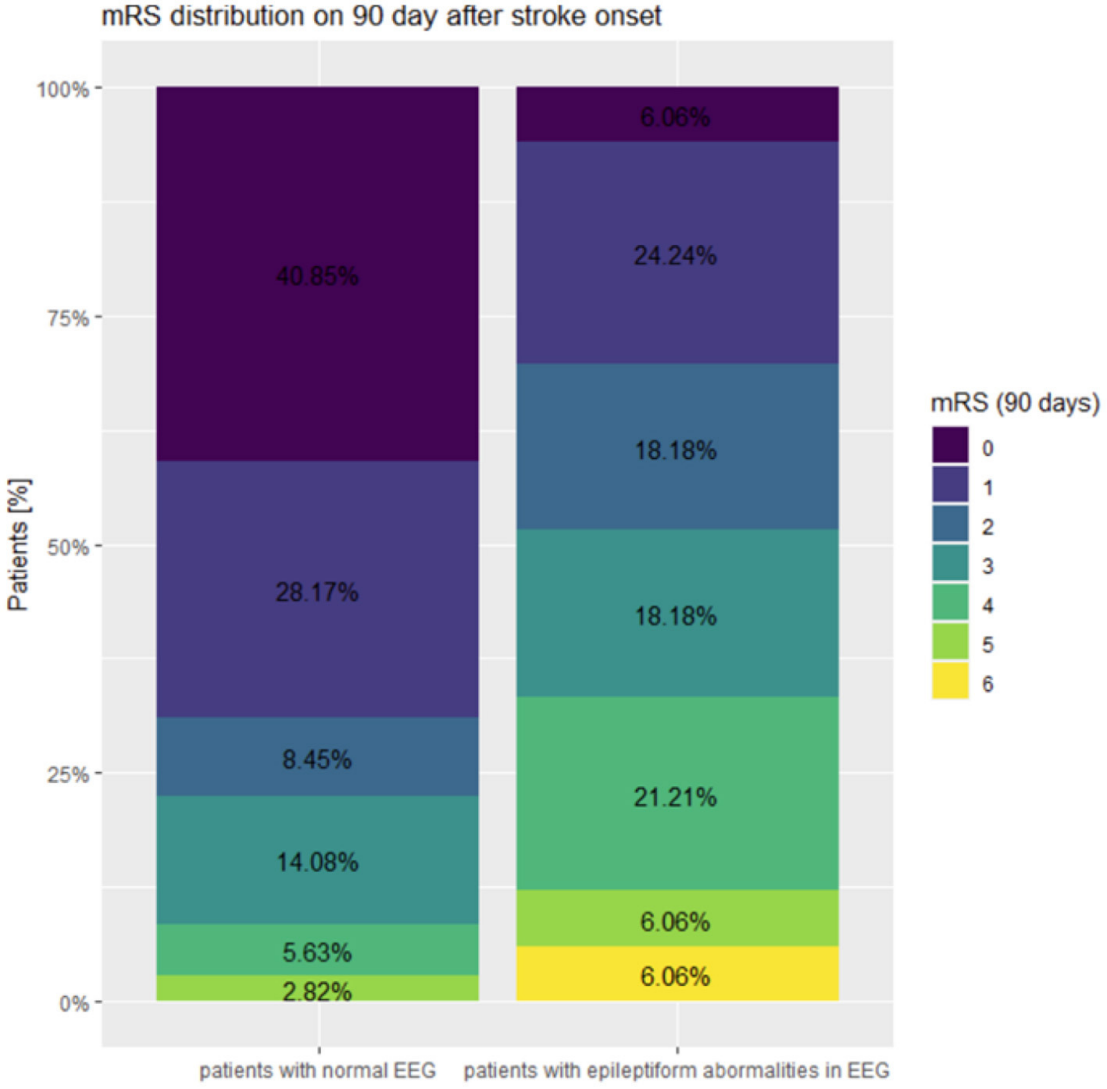
The distribution of mRS points on 90^th^ day after the onset of stroke in patients with normal EEG and in patients with epileptiform abnormalities.

The distribution of mRS points on 90^th^ day after the onset of stroke in patients with and without epileptiform abnormalieties is presented in Figure 1.

Two independent prognostic factors were identified for achieving a good functional status on the day of discharge: the neurological status as measured by the NIHSS on the first day and the absence of changes in the EEG results recorded in the hemisphere without stroke (Tables 4, 5).

**Table 4:** Ordinal regression analysis of the influence of selected parameters regarding good functional status (mRS 0-2) upon discharge.

**Coefficients**	**Adjusted OR**	**CI 95%**	***P*-value**
Age	0.96	(0.91–1.00)	0.08
Sex	1.75	(0.56–5.56)	0.33
NIHSS_1	0.77	(0.66–0.87)	<0.01
Stroke location^*^	1.07	(0.56–2.04)	0.81
OCSP	0.39	(0.10–1.28)	0.13
Therapy	0.90	(0.63–1.27)	0.56
EEG_stroke hemisphere	0.92	(0.60–1.43)	0.70
EEG_not-affected hemisphere	0.41	(0.16–0.95)	0.04

* Left or right hemisphere.

EEG, electroencephalography; NIHSS 1, National Institutes of Health Stroke Scale in the days 1–3 of stroke; OCSP, Oxfordshire Community Stroke Project.

**Table 5:** Ordinal regression analysis of the influence of clinical phenodata on mRS discharge after model selection using the AIC criterion and the area under the ROC curve.

**Coefficients**	**Adjusted OR**	**CI 95%**	***P*-value**
NIHSS_1	0.86	(0.81–0.92)	<0.01
EEG_not affected hemisphere	0.57	(0.36–0.87)	0.01

EEG, electroencephalography; NIHSS, National Institutes of Health Stroke Scale in the days 1–3 of stroke.

Only the neurological status on the first day of stroke according to the NIHSS score showed an independent effect on the functional status of patients on 90 days following stroke onset (Table 6).

**Table 6:** Ordinal regression analysis of the influence of selected parameters on mRS 90th day.

**Coefficients**	**Adjusted OR**	**CI 95%**	***P*-value**
Age	0.95	(0.89–1.00)	0.07
Sex	1.18	(0.32–4.39)	0.79
NIHSS_1	0.86	(0.77–0.96)	0.00
Stroke location^*^	0.75	(0.36–1.48)	0.42
OCSP	0.59	(0.17–1.95)	0.38
Therapy	1.01	(0.70–1.46)	0.93
EEG_stroke hemisphere	0.80	(0.52–1.26)	0.33
EEG_not-affected hemisphere	0.47	(0.21–1.02)	0.05

* Left or right hemisphere.

EEG, electroencephalography; NIHSS, National Institutes of Health Stroke Scale in the days 1–3 of stroke; OCSP, Oxfordshire Community Stroke Project.

The age-based analysis model (OR 0.981 CI 95% 0.959–1.001, *p* = 0.047), neurological status on day 1 (OR 0.884 CI 95% 0.82–0.942, *p* < 0.0001) and EEG recordings above the healthy hemisphere (OR 0.607 CI 95% 0.37–0.917, *p* = 0.028) had the highest prognostic value in terms of achieving good functional status on the 90^th^ day following stroke.

## Discussion

The results of this study indicate that as many as 40% of stroke patients without “clinical” epileptic seizures exhibit EEG abnormalities during the acute period of stroke. The changes recorded in the EEG have a varying morphology and are located both in the hemisphere with stroke focus and in the contralateral one. As many as 30% of the patients with changes in the EEG showed changes of varying morphology. For patients with abnormal EEG, a poorer neurological state was observed in the acute period of stroke, and a worse functional state was seen in the chronic period of stroke compared to patients without abnormal EEG readings. The results of the presented study align with the results obtained by other researchers (11).

Other authors have documented abnormal EEG recordings in 3–46% of stroke patients (10, 11, 14, 20–23). The discrepancies may result from the differing neurological statuses of the patients studied, various stroke types and locations (increased prevalence of changes in the EEG in cortical stroke). According to previous reports, both the extent of the stroke and the location of ischemic focus influence the risk of epileptic seizures (23). Most likely, it is the type and extent of ischemic lesions which affect the morphology of changes in the EEG recordings. PACI stroke patients predominated in the presented study. Here, the mean NIHSS score represents a moderate neurological deficit. The EEG results of our patients mostly showed sporadic discharges and generalized rhythmic delta activity.

There was no relationship found between therapy and changes in EEG in the presented study. Other authors have previously addressed the topic of the epileptogenic properties of rtPA. The properties of a rtPA-induced reperfusion/hyperperfusion were indicated by the results obtained in pre-clinical and clinical studies (stunned brain syndrome) (24). A recent meta-analysis and other studies indicate that rtPA is likely not associated with an increased risk of seizures (25–27).

Neurological status on the first and second day of stroke was significantly poorer in patients with abnormal EEG, probably due to the extent of brain damage they suffered.

Focal paroxysmal epileptiform abnormalities can not only result from acute ischemic brain damage, but can also secondarily contribute to damage escalation. Other authors have observed hypermetabolism in the neural tissue of patients with abnormal EEG, which resembles the hypermetabolism accompanying epileptic seizures (28). That phenomenon results in an increased local blood flow, higher perfusion pressure and reduced oxygen concentration (28, 29).

There has been a discussion about the importance of the metabolic mismatch between demand and supply in ischemic neural tissue. A reduced metabolic reserve in the injured brain combined with an increased metabolic demand induced by epileptiform abnormalities leads to secondary brain damage (22, 28–30). This may explain why patients with abnormal EEG readings have a decreased functional status even when no epileptic seizures were present. Some authors claim, that the sporadic epileptiform discharges because of their non-continuous nature don't cause metabolic stress according to the metabolic supply-demand mismatch hypothesis (10).

The presented study shows the relevance of the age and neurological status of patients on the first day of stroke to their post-stroke functional status in the acute and chronic period of stroke (31). These observations are consistent with reports by other authors. Bentes et al. found an association between abnormal EEG and post-stroke functional status in long-term follow-up (11).

We do not know the pre-stroke EEG recordings of our patients so the changes in bioelectrical brain activity may have been previously present in some patients due to clinically silent vascular lesions or neurodegenerative diseases. However, previous results from other authors indicate a weak association between generalized slowdown in bioelectric activity and the functional status of patients or epileptic seizures (32–34). Opposing results have also been published (35). Our study involved only patients with no history of epileptic seizures.

According to metabolic mismatch theory, it is unlikely that sporadic epileptiform discharges could induce metabolic stress. These do not seem to make any difference in terms of the condition of patients.

Interestingly, in the presented study, we also found that the absence of EEG abnormalities recorded above the non-stroke hemisphere in the acute period of stroke increased the chances of an improved functional status during the chronic phase of stroke. It is difficult to actually determine the value of this observation.

Previous studies using the functional magnetic resonance imaging to evaluate stroke patients have found that hemodynamic and functional changes occur in the hemisphere without the infarct focus. An explanation for this phenomenon is that it acts as a mechanism to reduce the effects of cerebral ischemia (36). Changes in EEG found above the “healthy” brain hemispheres of our patients may confirm the above interpretation. The changes recorded above the non-stroke brain hemisphere affected the prognosis in terms of the functional status of the patients on day 90.

The results of our study suggest that instrumental methods can possibly be used as prognostic tools. This could improve the value of clinimetric scales in this regard (37, 38).

Several studies have demonstrated that quantitative EEG indexes were of greater use in predicting the functional status rather than scales assessing clinical status (39, 40). Some authors showed that hyper-acute alterations of EEG parameters are highly related to the extent of hypoperfused tissue highlighting the value of quantitative EEG as a possible complementary tool in the evaluation of stroke severity and its potential role in acute ischemic stroke monitoring (41, 42). There have been reports suggesting that quantitative EEG indexes have a greater value in predicting post-stroke neurological status than the measurements of the focus size found *via* MRI (43).

Although there are well-defined risk factors for cardiovascular diseases they cannot, however explain the variability in presentation and prognosis. According to previous animal results, the myocardium infarction can cause changes in brain cortex activity. A recent study demonstrated significant increases in median band power of beta, theta and alpha brain waves during the experimental model of acute myocardium syndrome. It is possible that the ischemic phenomenon-related changes in cortex activity can independently influence the functional status of patients (44).

### Limitations

We did not analyze the potential association between infarct focus size on head CT/MRI, changes in EEG and patient functional status, which may form a limitation in this study.

## Conclusions

Abnormalities in EEG without clinical manifestation are present in 40% of patients with acute stroke.

During the acute stroke phase, abnormal bioelectric activity is recorded both in the affected and healthy brain hemispheres.

Changes in EEG in acute stroke are associated with a poorer neurological status in the first days of stroke.

Abnormal EEG in patients without epileptic seizures in acute stroke adversely affect the functional status of patients in the chronic period of stroke.

EEG readings in the acute period of stroke may prove to be a useful method in predicting the functional status of patients, this requires further confirmation in the studies to follow.

## Data availability statement

The original contributions presented in the study are included in the article/supplementary material, further inquiries can be directed to the corresponding author.

## Ethics statement

The studies involving human participants were reviewed and approved by Ethics Committee of the Silesian Medical University of Silesia in Katowice. The patients/participants provided their written informed consent to participate in this study.

## Author contributions

AL-B and MB contributed to conception and design of the study and wrote the manuscript. ŁB, AZ, EK, and BŁ-R organized the database. SS performed the statistical analysis. MD-S, AKa, AKr, and AC wrote sections of the manuscript. All authors contributed to manuscript revision, read, and approved the submitted version.
